# Callistemenonone A, a novel dearomatic dibenzofuran-type acylphloroglucinol with antimicrobial activity from *Callistemon viminalis*

**DOI:** 10.1038/s41598-017-02441-5

**Published:** 2017-05-24

**Authors:** Yu-Qing Xiang, Hong-Xin Liu, Li-Yun Zhao, Zhi-Fang Xu, Hai-Bo Tan, Sheng-Xiang Qiu

**Affiliations:** 1 Program for Natural Product Chemical Biology, Key Laboratory of Plant Resources Conservation and Sustainable Utilization, Guangdong Provincial Key Laboratory of Applied Botany, South China Botanical Garden, Chinese Academy of Sciences, Guangzhou, 510650 People’s Republic of China; 2 State Key Laboratory of Applied Microbiology Southern China, Guangdong Provincial Key Laboratory of Microbial Culture Collection and Application, Guangdong Open Laboratory of Applied Microbiology, Guangdong Institute of Microbiology, Guangzhou, 510070 People’s Republic of China; 3 Graduate University of Chinese Academy of Sciences, Beijing, 100049 People’s Republic of China

## Abstract

A new acylphloroglucinol with a novel architecture including an unprecedented dearomatic dibenzofuran core, named callistemenonone A (**1**), was isolated from the leaves of *Callistemon viminalis* (Myrtaceae). The structure was fully characterized on the basis of extensive spectroscopic analysis, including UV, HRESIMS, as well as 1D and 2D NMR spectral data (HSQC, HMBC, and ROESY). The deduced structure represents the first example of a natural dibenzofuran with two phenyl moieties coupling through tertiary hydroxy and ketal carbons. A plausible biogenetic pathway involving oxidative coupling and dearomatization as key steps is proposed to account for the biosynthesis of this novel class of dibenzofuran. Moreover, antimicrobial assays, in conjunction with the time-killing and biophysical studies, revealed that **1** exerted potent bactericidal activity against a panel of methicillin resistant pathogenic microbes with a unique mechanism.

## Introduction

The genus *Callistemon* belongs to the family Myrtaceae, and is comprised of over 30 species with most being woody, aromatic, and ornamental trees or shrubs endemic to Australia^1^. *Callistemon viminalis* (Sol. ex Gaertn,) G. Don (commonly known as red bottlebrush) is an Australian native green shrub with leaves and stems reputed to possess a variety of biological effects, including antidiabetic^2^, antimicrobial, anti-inflammatory, antistaphylococal, antithrombin^3–6^, and nematicidal, larvicidal, as well as pupicidal activities^7^. It was introduced into China some decades ago. Presently, it is widely cultivated in southern China as an ornamental tree and is adopted into Chinese folk medicine to treat colds and arthralgia^8^.

**Figure 1:**
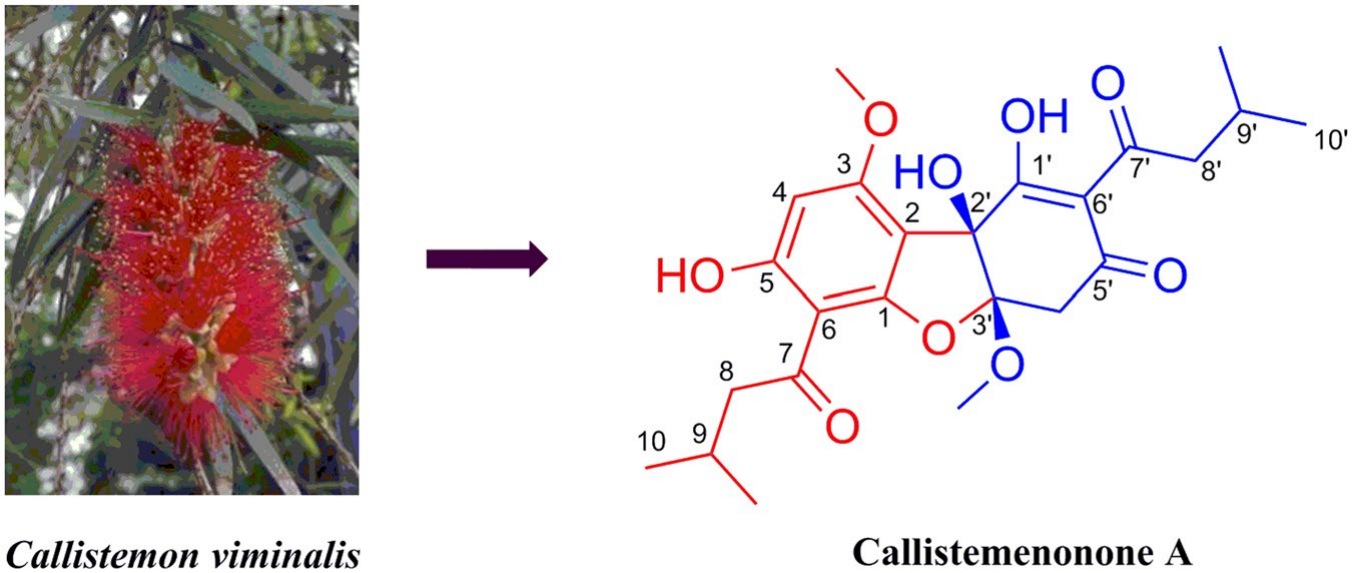
Structure of callistemenonone A (**1**).

Plants in the genus *Callistemon* are known for their enriched phloroglucinol content, which is responsible for a variety of the biological activities of these herbal medicines^4, 5^. Previous phytochemical studies on different parts of *C. viminalis* showed the presence of flavonoids^9^, triterpenoids^9^, tannins, and phloroglucinol derivatives^10, 11^. In a continuation of our efforts to discover new antibacterial constituents from the plants of family Myrtaceae^12–17^, the ethanolic extract of *C. viminalis* was found to exhibit *in vitro* antibacterial activity with a MIC value of 100 μg/mL against MRSA JCSC 2172. Antibactewrial activity guided isolation has now led to the isolation of a new potent (MIC 20 μg/mL, anti-MRSA JCSC 2172) acylphloroglucinol with an unprecedented dearomatic dibenzofuran skeleton, designated as callistemenonone A (**1**) (Fig. 1). Herein, the isolation, structural elucidation, bioassay, and antibacterial mechanism of **1** are described.

## Results and Discussion

*C. viminalis* leaves were extracted at room temperature with 95% EtOH, and the resulting pooled crude extract was concentrated under reduced pressure and then partitioned to afford *n*-hexane-soluble, EtOAc-soluble, and aqueous fractions. The *n*-hexane and EtOAc-soluble fraction were combined and subjected to repeated silica gel column chromatography to obtain **1** as a yellowish oil.

Compound **1** exhibited a deprotonated molecular ion at *m*/*z* 461.1819 (calcd *m*/*z* 461.1812) in the negative high-resolution electrospray ionization mass spectroscopy (HRESIMS) spectrum (HRESIMS and 1D and 2D spectra see supplementary information), consistent with the molecular formula C_24_H_29_O_9_, indicating nine indices of hydrogen deficiency. In good agreement with the molecular formula, inspection of the ^13^C NMR (Table 1) and HSQC spectra identified a total of 24 carbons, including four carbonyl (*δ*_C_ 191.1, 191.9, 200.1, 204.1), eight quaternary (*δ*_C_ 168.7, 162.0, 160.3, 110.3, 108.4, 105.2, 101.7, 82.8), three methylene (*δ*_C_ 51.7, 45.6, 42.2), three methine (*δ*_C_ 94.1, 26.5, 26.0), four methyl (*δ*_C_ 22.8, 22.6, 22.4, 22.3), and two methoxy (*δ*_C_ 55.9, 56.1) carbons. The ^1^H NMR data (Table 1) showed characteristic resonance signals for four methyl groups at *δ*_H_ 0.98 (H-10), 1.02 (H-11), 0.75 (H-10′), and 0.80 (H-11′), as well as two methoxy groups at *δ*_H_ 3.85 (3-OMe) and 3.59 (3′-OMe).

**Table 1:** ^1^H and ^13^C NMR data of **1** in CDCl_3_ (*δ* in ppm, *J* in Hz).

position	*δ*_C_ (125 MHz)	*δ*_H_ (500 MHz)	HMBC correlations
1	160.3 s		
2	105.2 s		
3	162.0 s		
4	94.1 d	6.02 (s)	C-2, C-3, C-5, C-6
5	168.7 s		
6	101.7 s		
7	204.1 s		
8	51.7 t	2.96 (dd, 7.0, 14.4), 2.72 (dd, 7.0, 14.4)	C-7, C-9, C-10, C-11
9	26.0 d	2.12 (m)	C-7, C-8, C-10, C-11
10	22.6 q	0.98 (d, 6.8)	C-8, C-9, C-11
11	22.8 q	1.02 (d, 6.8)	C-8, C-9, C-10
1′	191.1 s		
2′	82.8 s		
3′	108.4 s		
4′a 4′b	42.2 t	3.11 (d, 16.3), 3.26 (d, 16.3)	C-2′, C-3′, C-5′, C-6′
5′	191.9 s		
6′	110.3 s		
7′	200.1 s		
8′	45.6 t	2.77 (dd, 7.3, 13.4), 2.39 (dd, 7.3, 13.4)	C-7′, C-9′, C-10′, C-11′
9′	26.5 d	1.83 (m)	C-7′, C-8′, C-10′, C-11′
10′	22.3 q	0.75 (d, 6.8)	C-8′, C-9′, C-11′
11′	22.4 q	0.80 (d, 6.8)	C-8′, C-9′, C-10′
3-OMe	56.1 q	3.85 (s)	C-3
3′-OMe	55.9 q	3.59 (s)	C-3′
2-OH		4.60 (s)	
5-OH		13.7 (s)	C-4, C-5, C-6

**Figure 2:**
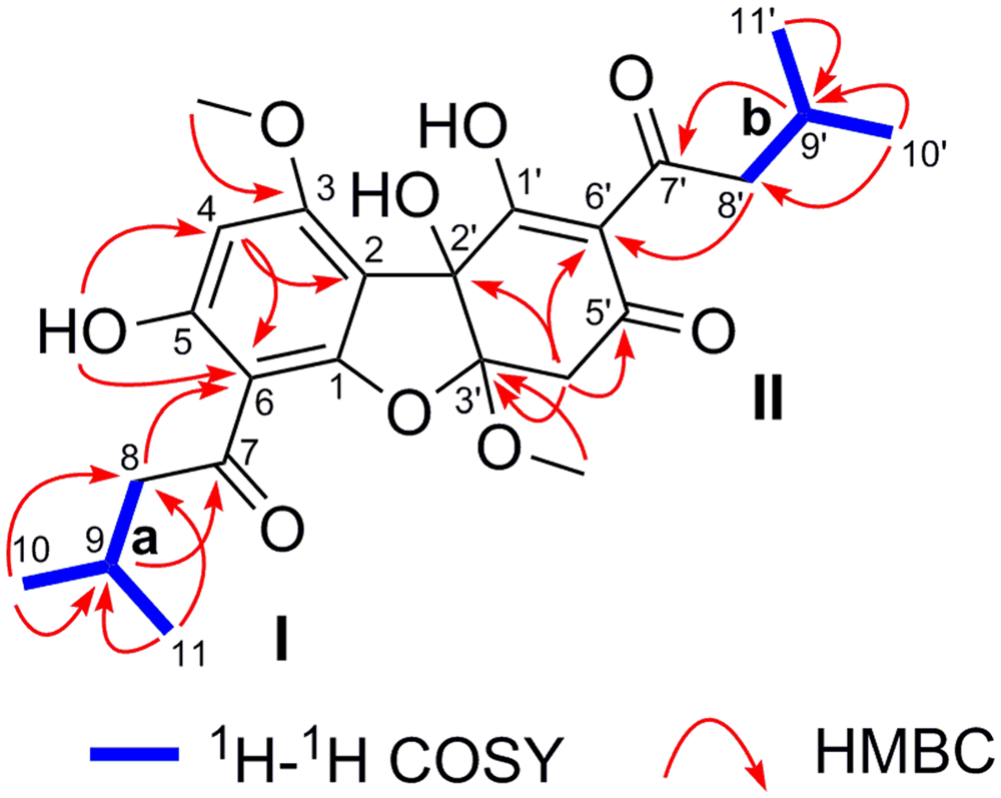
The key ^1^H-^1^H COSY and HMBC correlations (H → C) of **1**.

The ^1^H–^1^H COSY spectrum revealed the presence of two spin-coupling fragments (Fig. 2), namely, **a** (C-8/C-9/C-10/C-11) and **b** (C-8′/C-9′/C-10′/C-11′). The key HMBC cross-peaks (Fig. 2) observed in the coupling fragment **a** were H_3_-10 to C-8, C-9, and C-11; H_3_-11 to C-8, C-9, and C-10; H-9 to C-7; and H-8 to C-6. These correlations confirmed the presence of an isovaleryl group. Moreover, the HMBC correlations of the 5-OH to C-4, and C-6, of H-4 to C-2, and C-6, as well as of the 3-OMe to C-3 indicated the presence of a penta-substituted benzene moiety. Furthermore, the HMBC cross peak between H-8 to C-6 suggested a connection of the isovaleryl group to C-6 of the benzene moiety, which led to the construction of a typical phloroglucinol unit **I**, which was characteristically found in the genus *Callistemon* ^18, 19^. Similarly, the HMBC correlations of H_3_-10′ to C-8′, C-9′, and C-11′; of H_3_-11′ to C-8′, C-9′, and C-10′; of H-9′ to C-7′, and of H-8′ to C-6′, together with key HMBC correlations of H-4′ to C-3′, of C-2′ and C-6′, as well as of the 3′-OMe to C-3′, confirmed the other sub-structure portion as **II** (Fig. 2).

**Figure 3:**
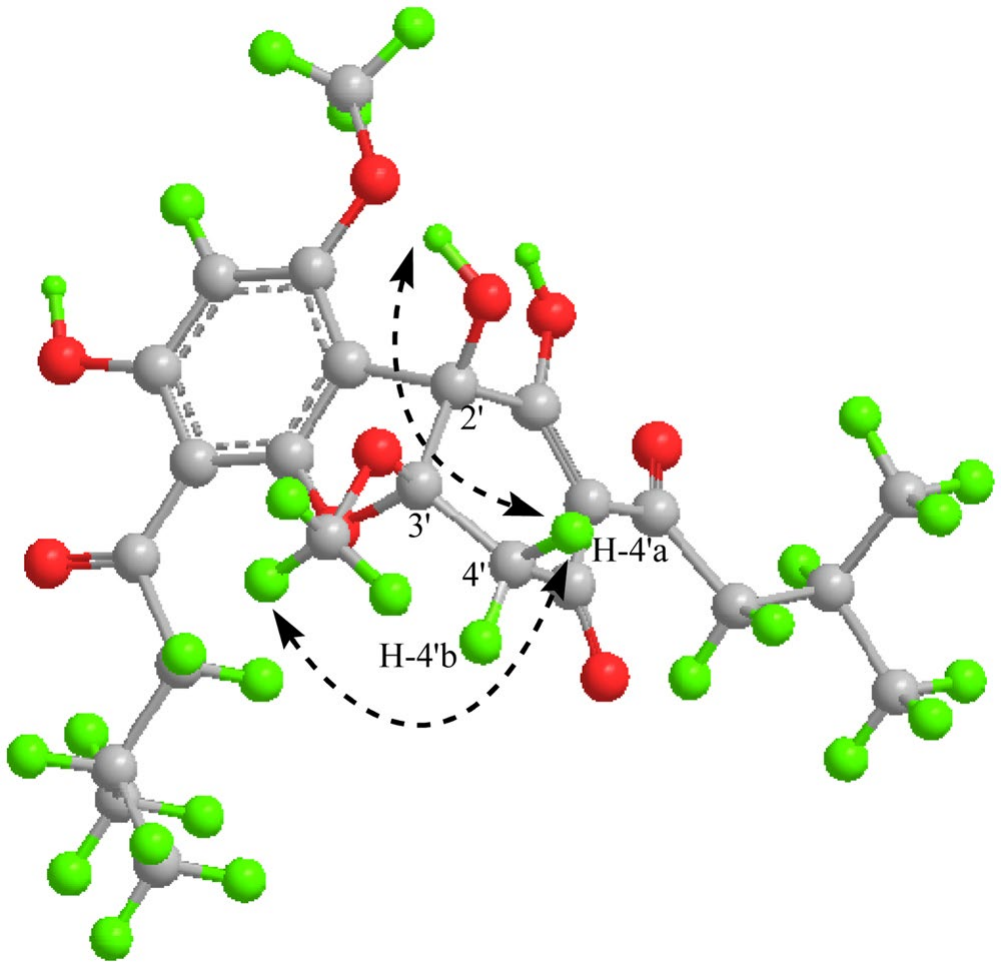
The key ROESY correlations (H → H) of **1**.

Although the HMBC experiment failed to provide direct and conclusive evidence for the linkage between the structural fragments **I** and **II** due to the absence of a suitable correlative proton, assembly of the two moieties was achieved through a connection of C-1-O-C-3′ which led to the presence of a furan ring and, together with portions **I** and **II**, accounted for the nine double bond equivalents. Compound **1** might be an equimolar mixture of the two enantiomers based on its zero optical rotation value^10^. The relative configuration of **1** was deduced from an analysis of the ROESY correlations (Fig. 3). The key ROE correlations between the 2′-OH/H-4a′ and the 3′-OMe/H-4′a indicated that the 2′-OH and 3′-OMe were cofacial. Therefore, the structure was determined as **1**, and given the trivial name callistemenonone A, representing the first member of a new family of phloroglucinol derivatives with an unprecedented dearomatic dibenzofuran core combining an *α*,*β*-triketone with a phloroglucinol unit.

**Figure 4:**
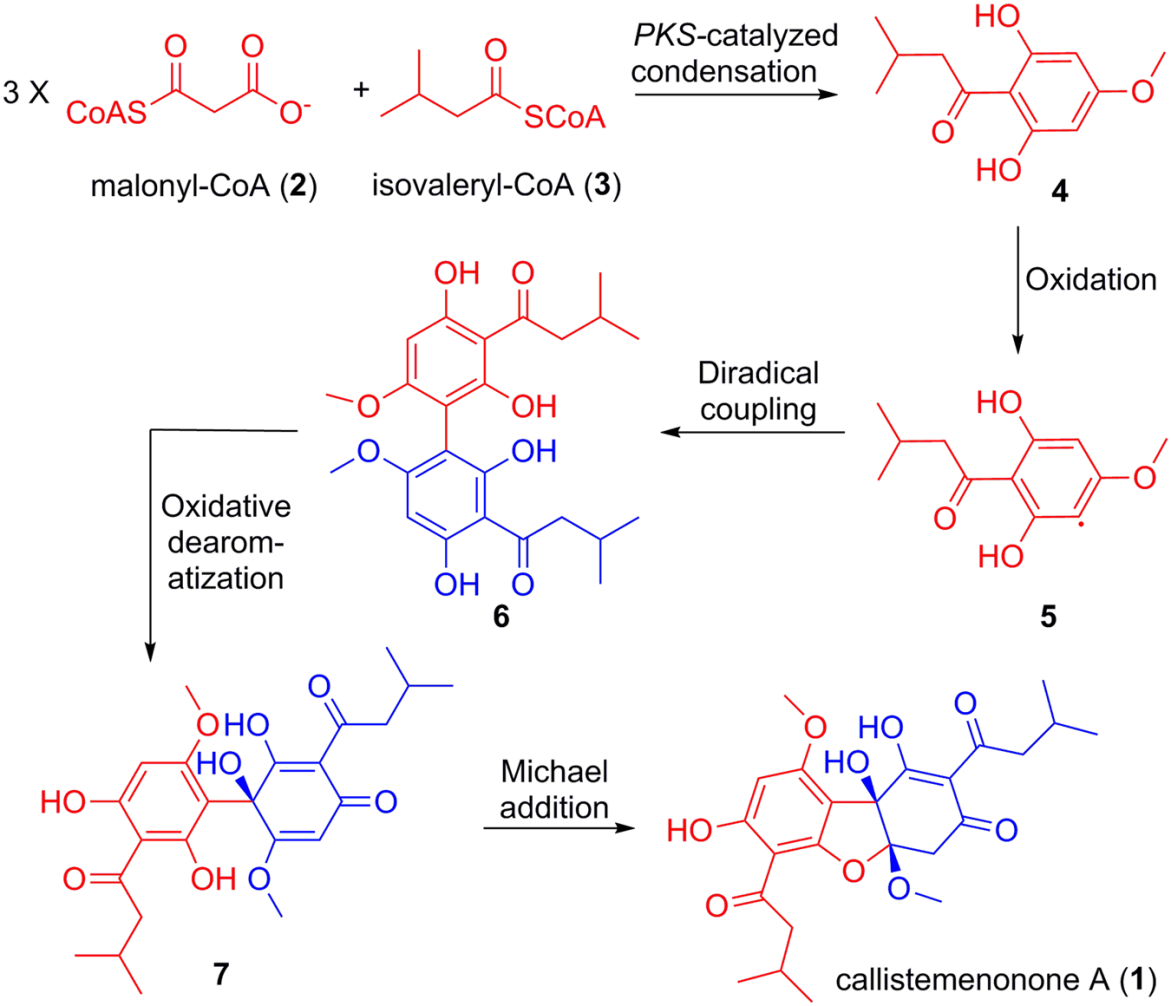
Proposed biogenesis of callistemenonone A (**1**).

A biotransformation pathway (Fig. 4) was proposed to account for the biogenesis of callistemenonone A (**1**). Briefly, isovaleryl-CoA (**2**) and malonyl-CoA (**3**) would undergo PKS (polyketide synthase)-catalyzed condensation to yield the acylphloroglucinol **4**, which has previously been reported from plants of the Myrtaceae family^11, 20, 21^. Oxidative coupling leads to the putative key precursor **6** through the radical intermediate **5**. Further oxidative dearomatization of **6** would generate the intermediate **7**, leading to construction of the unique tertiary hydroxyl carbon and the formation of the dibenzofuran architecture in callistemenonone A (**1**) through Michael addition.

The antimicrobial activity of **1** was evaluated towards a panel of Gram-positive bacteria, including *Staphylococcus aureus* strain (CMCC 26003), *Bacillus cereus* strain (CMCC 63302) and several strains of methicillin-resistant *S. aureus* MRSA JCSC 2172, MRSA JCSC 4469, and MRSA JCSC 4744 using standard MIC method and following the CLSI guidelines. As a result, compound **1** was shown to possess potent antibacterial activity against these Gram-positive bacteria with MICs and MBCs ranging from 5 to 80 μg/mL as presented in Table 2. The potency is 3–60 fold less than that the positive control substance vancomycin (Van). Callistemenonone A (**1**) was inactive towards the Gram-negative bacteria *Escherichia coli* (ATCC 8739), presumably due to its relative inability to penetrate the permeability barrier presented by the outer membrane and/or the action of efflux transporters.

**Table 2:** *In vitro* antibacterial activities of callistemenonone A (**1**) against bacteria strains.

Compd	MIC/MBC (μg/mL)					
	*S. aureus* CMCC 26003	*B. cereus* CMCC 63302	MRSA JCSC 2172	MRSA JCSC 4469	MRSA JCSC 4744	*E. coli* ATCC 8739
**1**	20/40	5/20	20/40	40/80	40/80	>400/ND
Van^a^	1.25/2.5	1.25/2.5	1.25/2.5	1.25/2.5	0.62/2.5	>400/ND

^a^Positive control; Van = vancomycin; ND: not determined.

**Figure 5:**
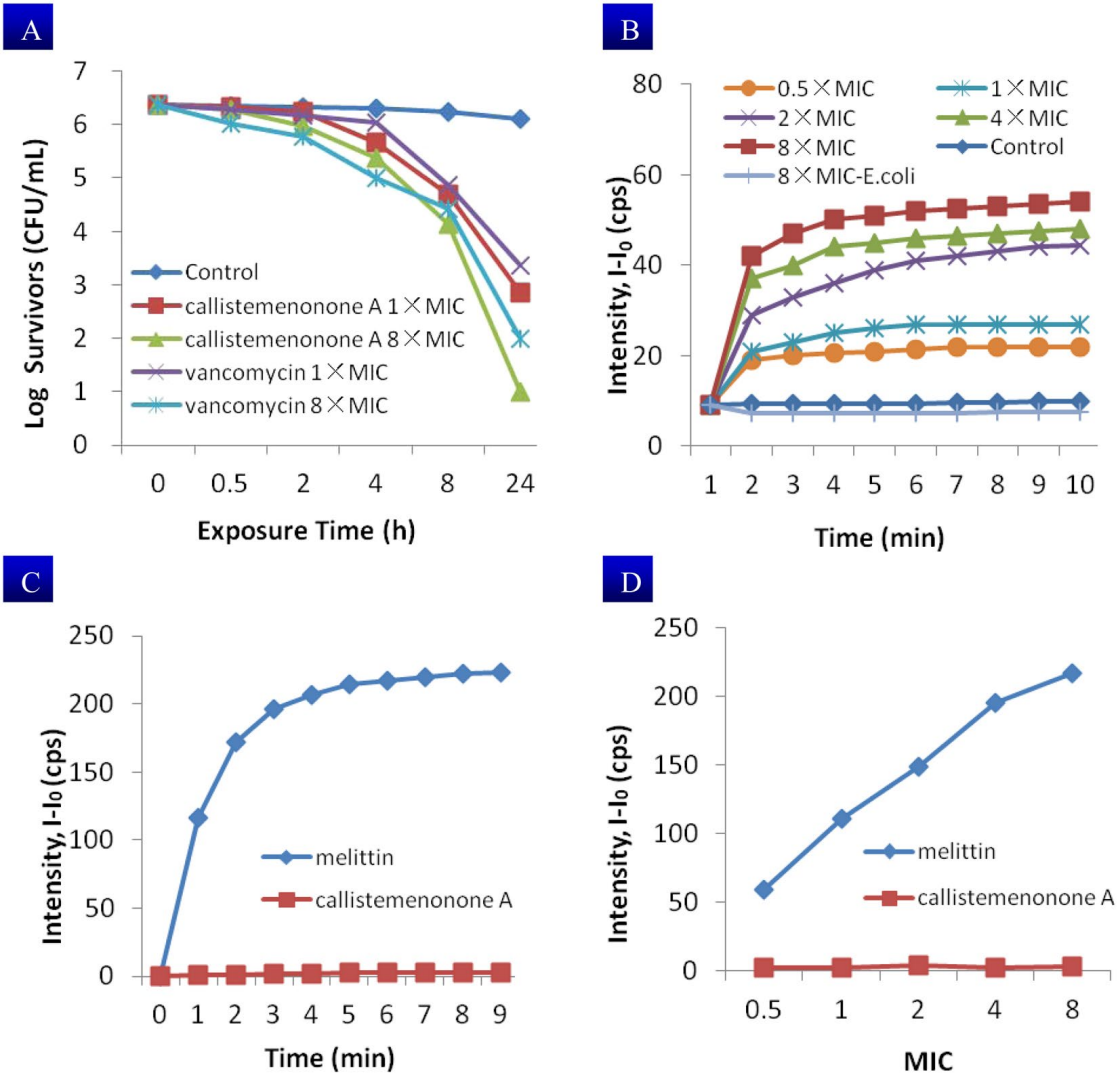
Antibacterial action of callistemenonone A (**1**). (**A**) Time-kill assay of MRSA (JCSC 2172) using **1** and vancomycin as control; (**B**) **1**-induced membrane depolarization of MRSA with different concentrations in the presence of DiSC3-5, DMSO was used as the untreated control with no effect on the depolarization, 8 × MIC concentration of **1** can not induce membrane depolarization of *E. coli*; (**C**) SYTOX green assay of MRSA with **1** (8 × MIC = 160 μg/mL) and melittin (10 μg/mL) as positive control; (**D**) SYTOX green assay after treating MRSA with different concentrations of **1** for 4 h and melittin as positive control. MIC values: callistemenonone A = 20 μg/mL, melittin = 1.25 μg/mL.

In order to understand the actions of callistemenonone A (**1**) at the morphologic level and to shed light on the mechanism underlying the antimicrobial activity against the clinical MRSA isolate JCSC 2172, a mechanistic investigation was initiated combining morphological, biochemical, and biophysical studies. As a result, time-kill kinetic experiments dosing with 1 × MIC revealed that **1** exhibited rapid, *in vitro* bactericidal activity (killing > 90% within 4 h) and complete bactericidal effect, with a sharp reduction in CFU of 99.99%, after 24 h exposure (Fig. 5A). However, in contrast to vancomycin, concentration-dependent experiment of **1** with 8 × MIC did not illustrate a dose-response effect.

Further biophysical studies using fluorescence probes for membrane potential were performed to determine whether **1** caused cytoplasm membrane depolarization^22–24^. As shown in Fig. 5B, after treating the MRSA JCSC 2172 cells with serial concentrations of **1** (0.5 × MIC, 1 × MIC, 2 × MIC, 4 × MIC, 8 × MIC), a sharp increase in the fluorescence was observed, suggesting that the favorable bactericidal action of **1** could be attributed to a significant dissipation of the membrane potential due to depolarization. However, the SYTOX green assay indicated that the bactericidal action of **1** was independent of membrane disruption^25, 26^, because there was no obvious increase of fluorescence detected when the MRSA suspension was treated even with 8 × MIC of **1** for up to 4 h. In contrast, the positive control melittin caused an unambiguous increase in fluorescence within 60 s (Fig. 5C) and in the whole concentrations range from 0.5 to 8 MIC after 4 h (Fig. 5D). These results were a strong indication that the bactericidal action of **1** was independent of membrane disruption.

**Figure 6:**
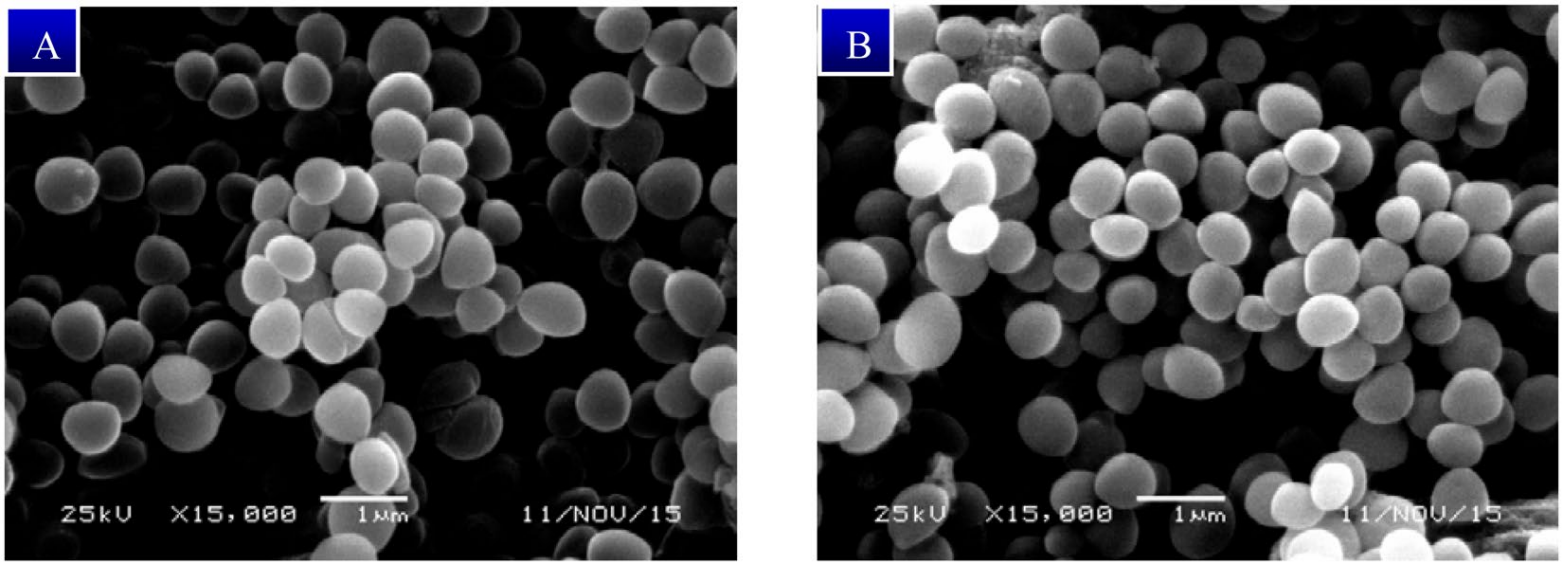
Scanning electron microscopy showed that callistemenonone A (**1**) induced no cell lysis and membrane disruption. (**A**) MRSA (JCSC 2172) incubated with callistemenonone A (**1**) (160 μg/mL, 8 × MIC) after 4 h; (**B**) Untreated MRSA (JCSC 2172).

Moreover, the aforementioned results were good in agreement with the morphological study using scanning electron microscopy, wherein **1** failed to disrupt the cytoplasmic membrane structure of MRSA JCSC 2172 cells following treatment with 8 × MIC of **1** for 4 h. As shown in Fig. 6, the morphology of the treated cells remained intact with smooth spheres comparable to those of the untreated control cells. Based on the results of membrane depolarization and membrane disruption assay, in conjunction with the time-killing study (killing > 90% after 4 h), it can be concluded that callistemenonone A (**1**) acts rapidly as a bactericidal antibiotic by disturbing the bacterial membrane potential without causing membrane disruption.

Melittin, the positive control used in several experiments, is a lytic and highly toxic peptide. It could be interesting to see if melittin and **1** in combination can lead to synergize the potency of 1 without undue toxicity from melittin. The synergism of **1** and melittin was investigated according to the Bliss independence model. Synergy value (0.025) was calculated using the formula as mentioned in Materials and Methods, this synergy value (0.025) demonstrated a very little synergistic relationship between **1** and melittin against MRSA.

In summary, these experiments have described herein the isolation, structural elucidation, and biological evaluation of a novel acylphloroglucinol named callistemenonone A (**1**), which was isolated from the leaves of *Callistemon viminalis* (Myrtaceae). The metabolite represents a new type of dimeric acylphloroglucinol with an unprecedented dearomatic dibenzofuran core which is biogenetically formed through tertiary hydroxy and ketal carbons. The oxidative coupling reaction and dearomatization were proposed to be key biotransformation steps for its biogenesis. The investigation of antibacterial mechanism, in conjunction with the time-killing study, revealed that **1** acted rapidly as a bactericidal agent by disturbing the bacterial membrane potential without causing membrane disruption. This represents a very favorable bactericidal mechanism which differs from any other antibiotic agent available either in the clinic or in development, rendering it a very promising candidate for further extensive investigation through medicinal chemistry, with an aim towards new antibiotic drug discovery.

## Experimental

### General procedures

Optical rotation was measured on a Perkin-Elmer 341 polarimeter (Perkin-Elmer, Boston, MA, USA). UV spectra were recorded in MeOH on a Perkin-Elmer Lambda 35 UV–vis spectrophotometer (Perkin-Elmer, Boston, MA, USA). 1D and 2D NMR spectra were recorded on a Bruker Advance-500 spectrometer with TMS as internal standard (Bruker BioSpin AG, Fällanden, Switzerland). HRESIMS data were obtained on a Bruker Bio TOF IIIQ mass spectrometer (Bruker Daltonics, Billerica, MA, USA). All solvents were analytical grade (Shanghai Chemical Plant, Shanghai, China). Silica gel (200–300 mesh) was used for column chromatography, and precoated silica gel GF_254_ plates (Qingdao Haiyang Chemical Plant, Qingdao, China) were used for TLC analysis. C_18_ reversed-phase silica gel (150–200 mesh, Merck), MCI gel (CHP20P, 75–150 μm, Mitsubishi Chemical Industries Ltd.), and Sephadex LH-20 gel (Amersham Biosciences) were also used for column chromatography. TLC spots were visualized under UV light and by dipping into 5% H_2_SO_4_ in alcohol, followed by heating. In the biological and biophysical experiments, the solvent used to dissolve **1** were DMSO.

### Plant material

The leaves of *C. viminalis* were collected from the South China Botanical Garden, Chinese Academy of Sciences in March 2015 and identified by Dr. Yun-Fei Deng of SCBG. A voucher specimen was deposited at the Laboratory of Natural Product Chemistry Biology, SCBG.

### Extraction and isolation

The chopped fresh leaves of *C. viminalis* (15.0 kg) were extracted with 95% Etch (30 L × 3) at room temperature for 24 hours. The pooled solvents were evaporated under reduced pressure to yield a brown syrupy residue (3.0 kg), which was suspended in H_2_O (3 L) and extracted successively with *n*-hexane (3 L × 3) and EtOAc (3 L × 3) to afford *n-*hexane- and EtOAc-soluble fractions. Most of spots of the two parts were similarity, which were detected by Thin layer chromatiography (TLC). So *n-*hexane- and EtOAc-soluble fractions (2.2 kg) were combined and subjected to silica gel column chromatography eluting with *n-*hexane/EtOAc with increasing polarity from 20:1 → 0:1 to yield six sub-fractions (A-F). Sub-fraction C (32.0 g) was chromatographed on a silica gel column with a gradient of CHCl_3_/MeOH (100:1 to 20:1) to yield three fractions (C_1_-C_3_). Sub-fraction C_2_ (3 g) was subjected to RP-C_18_ column chromatography (diameter: 60 cm, length: 254 mm; particle size: 150–200 mesh) and eluted with MeOH/H_2_O (90:10, 95:5, 100:0) to obtain three further sub-fractions (C_21_ to C_23_). Sub-fraction C_21_ (400 mg) was subjected to CC on Sephadex LH-20 (CHCl_3_/MeOH, 1:1, v/v) and further purified by repeated silica gel column chromatography (*n-*hexane/EtOAc, 20:1 → 1:1) to yield **1** (25 mg).

Callistemenonone A (**1**): Yellowish oil; [*α*]^20^_D_ = 0 (*c* 0.20, MeOH). UV (MeOH) *λ*_max_/nm (log *ε*) 238.7 (0.85), 281.0 (0.98); ^1^H (500 MHz) and ^13^C (125 MHz) NMR data, see Table 1; negative ESIMS *m*/*z* 461 [M − H]^−^; HRESIMS *m*/*z* 461.1819 [M − H]^−^ (for C_24_H_29_O_9_, calcd. 461.1810).

### Antibacterial assay

Compounds were dissolved in DMSO. The minimum inhibitory concentration (MIC) evaluation was carried out in 96-well plate according to the standard microdilution method. The minimum bactericidal concentration (MBC) was determined by sub-culturing 100 μL bacterial suspensions from the wells on the TSA plates. The plates were incubated for 24 h before the MBCs were determined. The MBC was categorized as the concentration where ≥ 99.9% reduction in bacterial cell count was observed. Test strains were *S. aureus* (CMCC 26003), *B. cereus* (CMCC 63302), and *E. coli* (ATCC 8739) which were purchased from Guangdong Microbiology Culture Center (Guangzhou, China). MRSA (JCSC 2172), MRSA (JCSC 4469), MRSA (JCSC 4744) were provided by T. Ito and K. Hiramatsu.

### Time killing assay

An overnight culture of cells (MRSA JCSC 2172) was adjusted in 0.85% NaCl buffer to obtain a bacterial suspension with 10^6^ to 10^7^ CFU/mL The inoculate were treated with various concentrations (1× and 8 × MIC) of callistemenonone A (**1**) and vancomycin. The mixtures were incubated at 37 °C. Culture aliquots were removed at 0 h, 0.5 h, 2 h, 4 h, 8 h, and 24 h, then they were serially diluted to 10^0^–10^6^ times and 100 μL 10-fold serially diluted suspensions were plated on TSA plates. The TSA plates were incubated at 37 °C for 24 h, colonies were counted to calculate surviving cfu/mL. Bactericidal activity was defined as a ≥ 3-log10 CFU/mL decrease, in comparison with the baseline, after 24 h of incubation^27^.

### Cytoplasmic membrane depolarization Assay

The effect of callistemenonone A on the membrane potential of MRSA JCSC 2172 cells was investigated using a modified version of the method described by Wu and Hancock^28^. Briefly, cultures of MRSA (JCSC 2172) were grown to exponential phase and then harvested. Bacteria were suspended and washed with buffer solution (5 mM HEPES at pH 7) until an optical density OD 0.1 at 600 nm (OD_600_) was obtained. The cell suspension was then incubated with 0.4 μM 3,3-dipropylthiacarbocyanine (DiSC_3_-5) for 20 min at 37 °C with shaking. Then, 100 mM KCl was added to the buffer to balance the chemical potential of K^+^ inside and outside the cells. The desired concentration of callistemenonone A (**1**) was added into a stirred cuvette, and the change in fluorescence was monitored at an excitation wavelength of 660 nm and an emission wavelength of 675 nm. DMSO and vancomycin was used as the negative controls with no effect on the depolarization. Experiments were repeated at least three times and found to be reproducible. Data from an individual experiment are presented.

### SYTOX green assay

The protocol was based on the method of Rathinakumar *et al*.^29^. Briefly, MRSA (JCSC 2172) cells were suspended in 0.85% NaCl buffer until an OD_600_ of 0.2 was obtained. The bacterial suspension was incubated with serial concentrations of callistemenonone A (**1**), respectively for 4 h, and then incubated with 3 μM of SYTOX green for 5 min. The fluorescence was measured at an excitation wavelength of 504 nm and an emission wavelength of 523 nm. Melittin, a cell lytic factor, was used as the positive control. Experiments were repeated at least three times and found to be reproducible. Data from an individual experiment are presented.

### Scanning electron microscopy

For the observations using scanning electron microscopy, exponential-phase bacteria were treated with the compound at 8 × MIC for 4 h at 37 °C. The cells were washed twice, suspended in PBS, and prefixed in 0.1 M phosphate buffer (pH 7.2) containing 2% glutaraldehyde and 2.5% paraformaldehyde overnight. After washing six times with 0.1 M phosphate buffer, the samples were post-fixed in 1% osmium tetroxide for 2 h. After washing another three times with 0.1 M phosphate buffer, the samples were dehydrated through a graded ethanol series and subjected to freeze-drying (JFD-310, JEOL, Tokyo, Japan). Samples were then mounted on stubs and coated with gold in a sputter coater (JFC-1600, JEOL, Tokyo, Japan), and then examined and photographed under a scanning electron microscope (JSM-6360LV, JEOL, Tokyo, Japan).

### Synergistic assay

The synergy of **1** in combination with melittin was investigated in vitro against MRSA2172 using the Bliss independence model as described in previous reports^30^. Briefly, bacterial strains were incubated with a sub-inhibitory concentration of simvastatin and control antimicrobials for 12 h and the degree of synergy was calculated using the formula: Synergy (S) was calculated using the formula: S = (f_A0_/f_00_)(f_0B_/f_00_) − (f_AB_/f_00_).The parameter f_AB_ refers to the optical density of the bacteria grown in the presence of **1** and melittin; parameters f_A0_ and f_0B_ refer to the bacterial growth rate in the presence of **1** alone and melittin alone, respectively; the parameter f_00_ refers to the bacterial growth in the absence of drugs. Degree of synergy (S) values corresponds to the following cut-offs: Zero indicates neutral, values above zero (positive value) represents synergism, and values below zero (negative values) correspond to antagonism. Drug combinations with higher positive value represents high degree of synergism.

## Electronic supplementary material

Supplementary information
