# Supplementary material for: Callistemenonone A, a novel dearomatic dibenzofuran-type acylphloroglucinol with antimicrobial activity from Callistemon viminalis
Source: Sci Rep. 2017 May 24;7:2363. doi: 10.1038/s41598-017-02441-5 (PMC5443826; doi:10.1038/s41598-017-02441-5)
Supplement: Supplementary file 1 — Supplementary information [file 41598_2017_2441_MOESM1_ESM.pdf]

Supplementary Information

Callistemenonone A, a novel dearomatic dibenzofuran-type acylphloroglucinol with antimicrobial activity from *Callistemon viminalis*

Yu-Qing Xiang[1,†], Hong-Xin Liu[1,2,†], Li-Yun Zhao[1,3,†], Zhi-Fang Xu[1], Hai-Bo Tan[1,*] & Sheng-Xiang Qiu[1,*]

[1]Program for Natural Product Chemical Biology, Key Laboratory of Plant Resources Conservation and Sustainable Utilization, Guangdong Provincial Key Laboratory of Applied Botany, South China Botanical Garden, Chinese Academy of Sciences, Guangzhou, 510650, People's Republic of China.

[2]State Key Laboratory of Applied Microbiology Southern China, Guangdong Provincial Key Laboratory of Microbial Culture Collection and Application, Guangdong Open Laboratory of Applied Microbiology, Guangdong Institute of Microbiology, Guangzhou 510070, People's Republic of China

[3]Graduate University of Chinese Academy of Sciences, Beijing 100049, People's Republic of China

[†]These authors contributed equally to this work

Contents

Figure S1. HRESIMS spectrum of compound **1**.

Figure S2. ^{1}H NMR spectrum (500 MHz, $CDCl_3$) of compound **1**.

Figure S3. ^{13}C NMR spectrum (125 MHz, $CDCl_3$) of compound **1**.

Figure S4. COSY spectrum of compound **1**.

Figure S5. HSQC spectrum of compound **1**.

Figure S6. HMBC spectrum of compound **1**.

Figure S7. NOESY spectrum of compound **1**.

Figure S8. UV spectrum of compound **1**.

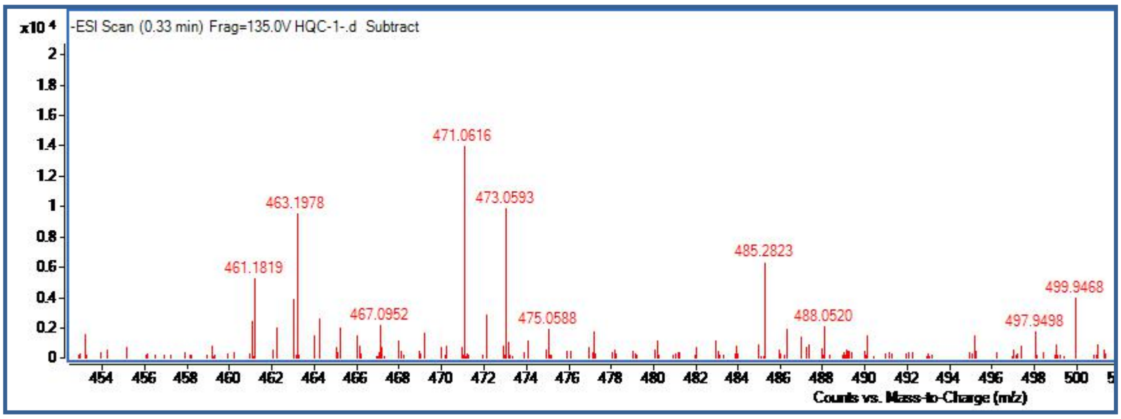

negative

Species, Calc m/z, Diff (ppm), Defect
(M-H)-, 461.1817, ,0.1817
M, 462.189, ,0.189
(M+Cl)-,497.1584, ,0.1584
(M+COOH)-,507.1872, ,0.1872
(M+CH_3COOH)-,522.2107, ,0.2107

Figure S1. HRESIMS spectrum of compound **1**.

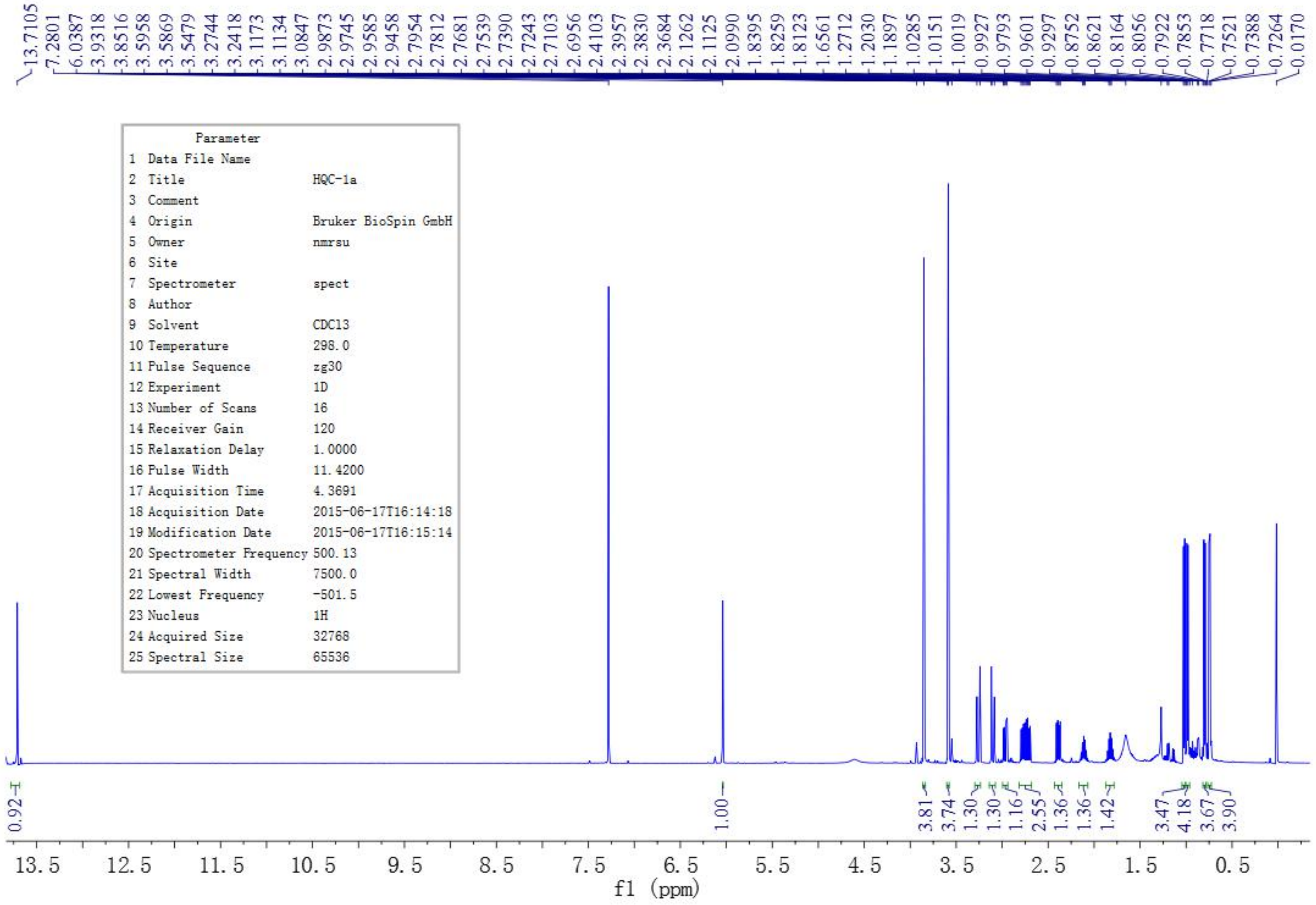

Figure S2. ^{1}H NMR spectrum (500 MHz, $CDCl_3$) of compound **1**.

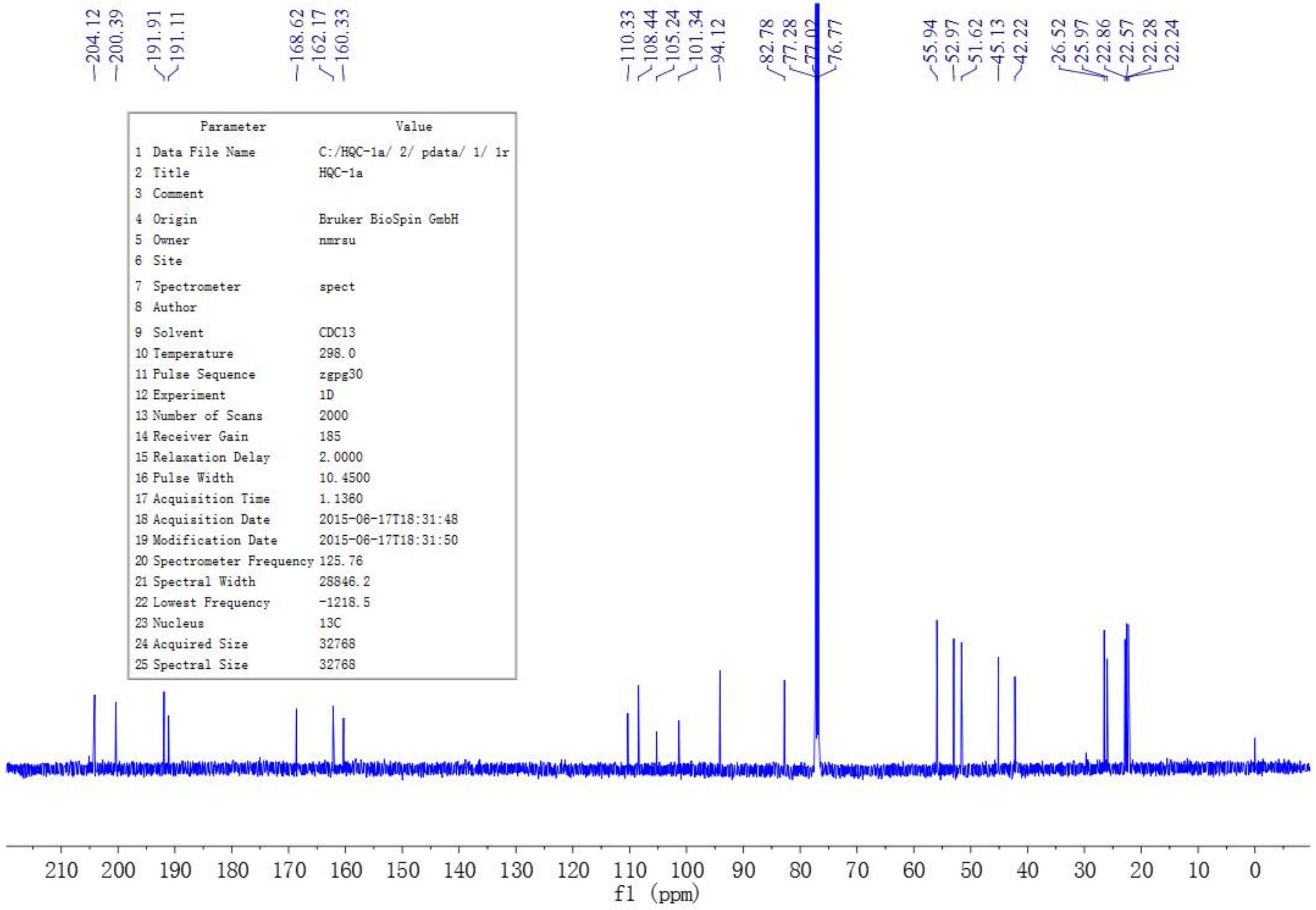

Figure S3. ^{13}C NMR spectrum (125 MHz, $CDCl_3$) of compound **1**.

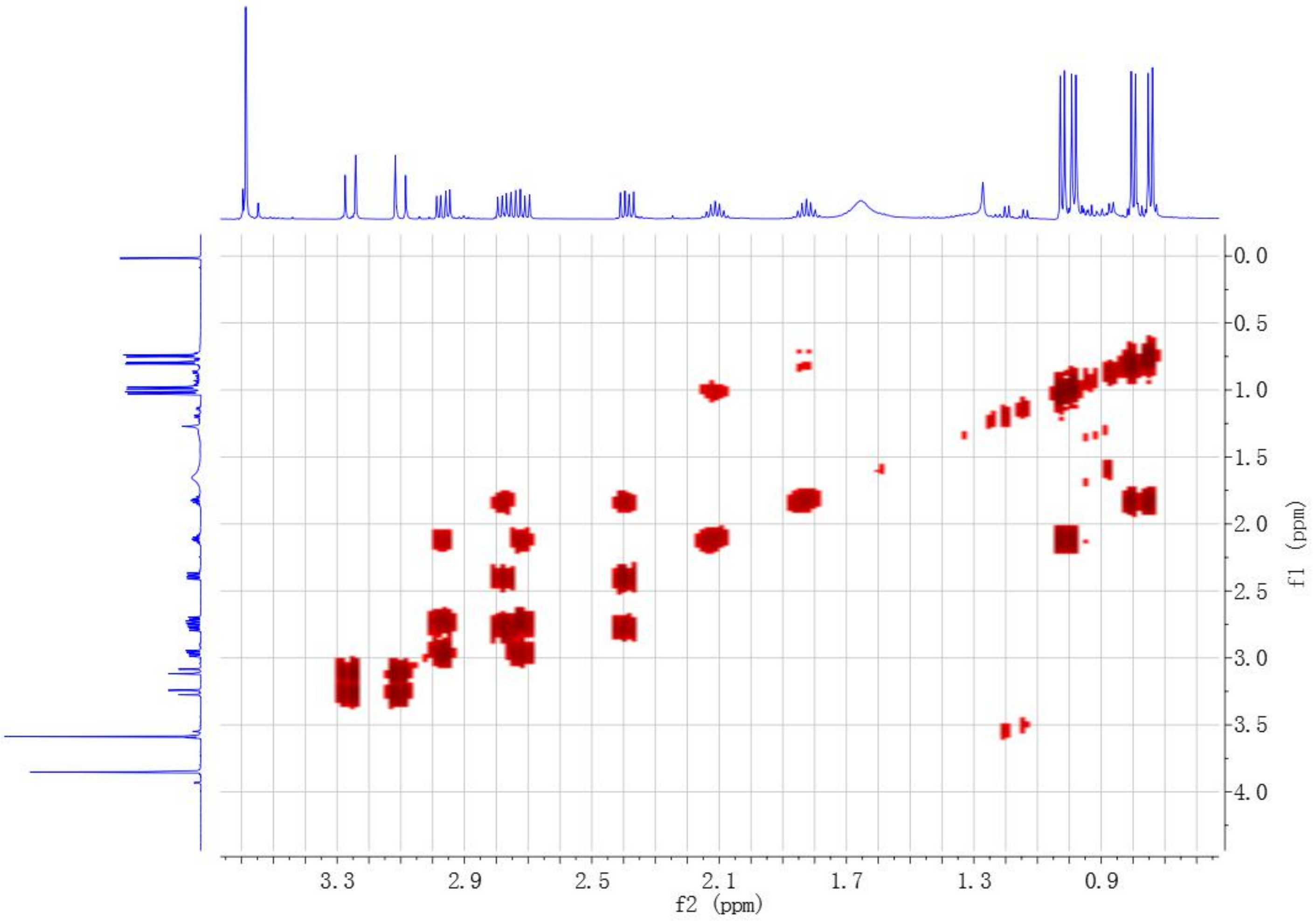

	Parameter	Value (f2, f1)
1	Data File Name	C:/HQC-1a/ 3/ pdata/ 1/ 2rr
2	Title	HQC-1a
3	Comment	
4	Origin	Bruker BioSpin GmbH
5	Owner	nmrsu
6	Site	
7	Spectrometer	spect
8	Author	
9	Solvent	CDCl3
10	Temperature	298.0
11	Pulse Sequence	cosygpmfqf
12	Experiment	2D-COSY
13	Number of Scans	8
14	Receiver Gain	185
15	Relaxation Delay	2.0221
16	Pulse Width	11.4200
17	Acquisition Time	0.1311
18	Acquisition Date	2015-06-17T18:33:04
19	Modification Date	2015-06-17T19:09:52
20	Spectrometer Frequency	(500.13, 500.13)
21	Spectral Width	(7812.5, 7812.5)
22	Lowest Frequency	(-441.1, -441.1)
23	Nucleus	(1H, 1H)
24	Acquired Size	(1024, 128)
25	Spectral Size	(1024, 1024)

Figure S4. COSY spectrum (125 MHz, $CDCl_3$) of compound **1**.

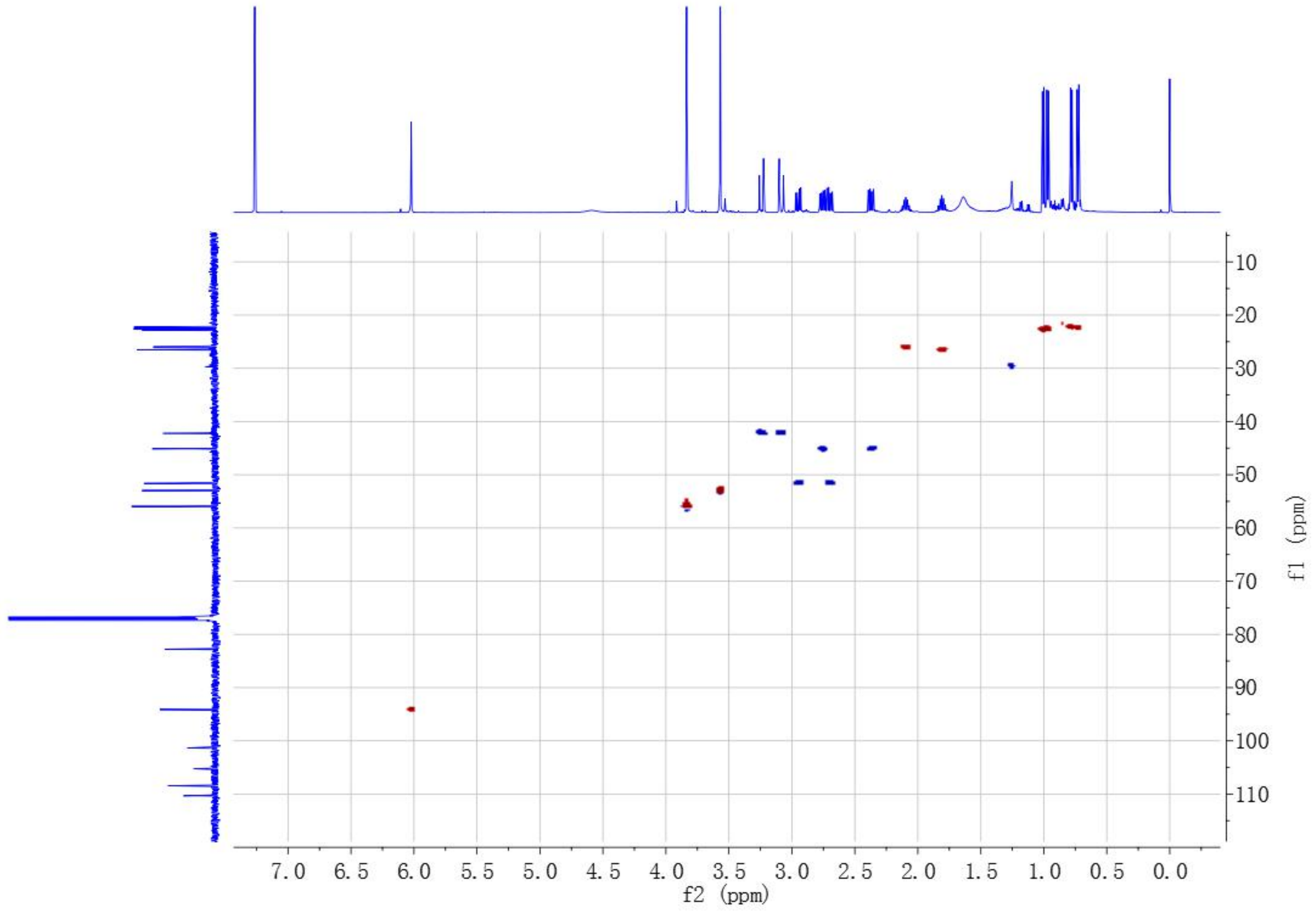

	Parameter	Value (f2, f1)
1	Data File Name	C:/HQC-1a/ 4/ pdata/ 1/ 2rr
2	Title	HQC-1a
3	Comment	
4	Origin	Bruker BioSpin GmbH
5	Owner	nmrsu
6	Site	
7	Spectrometer	spect
8	Author	
9	Solvent	CDCl3
10	Temperature	298.0
11	Pulse Sequence	hsqcedetgp
12	Experiment	2D-HSQC-EDITED
13	Number of Scans	4
14	Receiver Gain	185
15	Relaxation Delay	1.5154
16	Pulse Width	11.4200
17	Acquisition Time	0.0635
18	Acquisition Date	2015-06-17T19:11:22
19	Modification Date	2015-06-17T19:38:38
20	Spectrometer Frequency	(500.13, 125.76)
21	Spectral Width	(8064.5, 20833.3)
22	Lowest Frequency	(-683.9, -1037.0)
23	Nucleus	(1H, 13C)
24	Acquired Size	(512, 256)
25	Spectral Size	(1024, 1024)

Figure S5. HSQC spectrum of compound **1**.

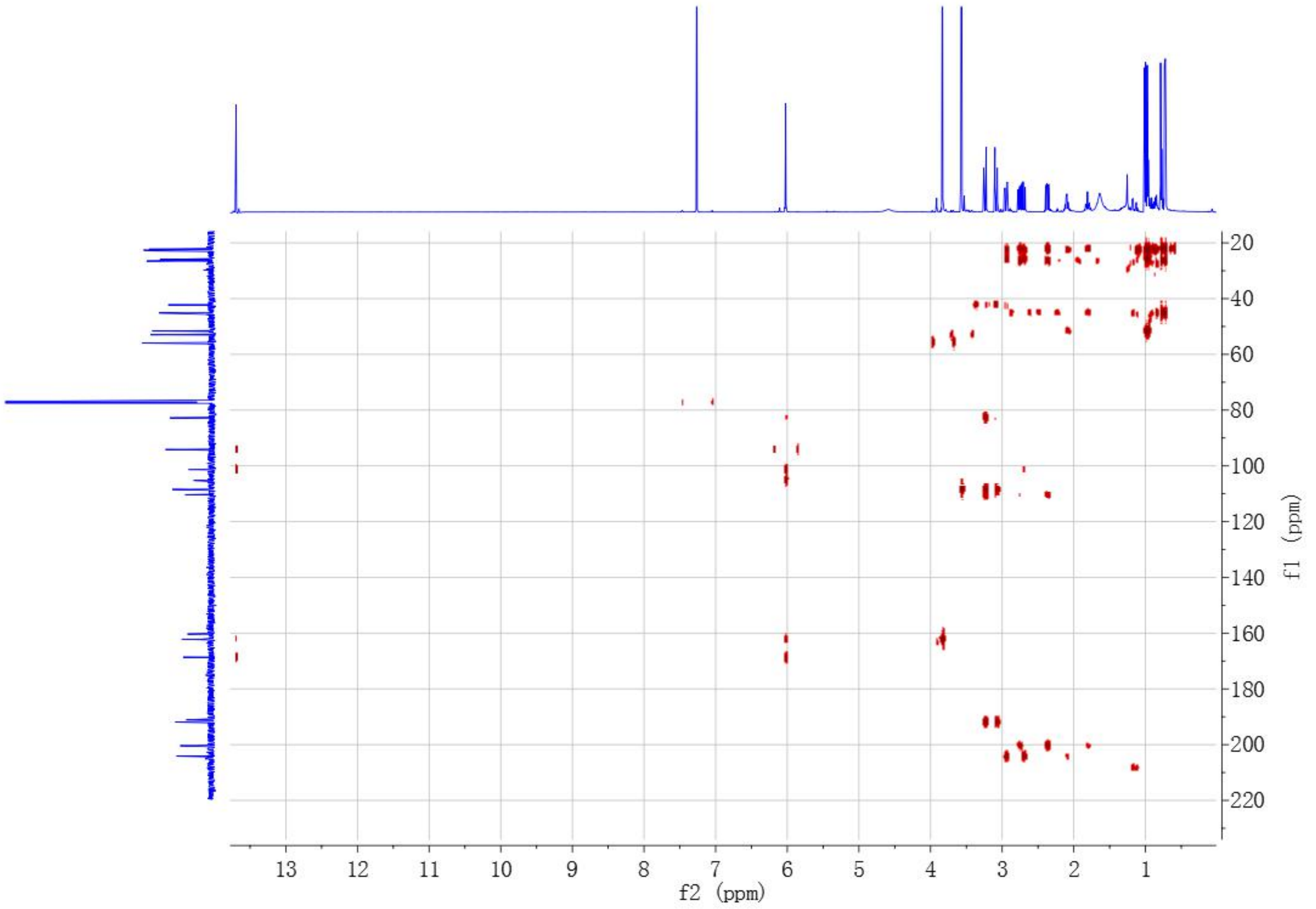

Parameter	Value (f2, f1)
1 Data File Name	C:/HQC-1a/ 5/ pdata/ 1/ 2rr
2 Title	HQC-1a
3 Comment	
4 Origin	Bruker BioSpin GmbH
5 Owner	nmrsu
6 Site	
7 Spectrometer	spect
8 Author	
9 Solvent	CDCl3
10 Temperature	298.0
11 Pulse Sequence	hmbcgpndqf
12 Experiment	2D-HMBC
13 Number of Scans	32
14 Receiver Gain	185
15 Relaxation Delay	1.5000
16 Pulse Width	11.4200
17 Acquisition Time	0.2925
18 Acquisition Date	2015-06-17T19:41:02
19 Modification Date	2015-06-17T21:47:20
20 Spectrometer Frequency	(500.13, 125.76)
21 Spectral Width	(7002.8, 30120.5)
22 Lowest Frequency	(-112.2, -220.8)
23 Nucleus	(1H, 13C)
24 Acquired Size	(2048, 128)
25 Spectral Size	(1024, 1024)

Figure S6. HMBC spectrum of compound **1**.

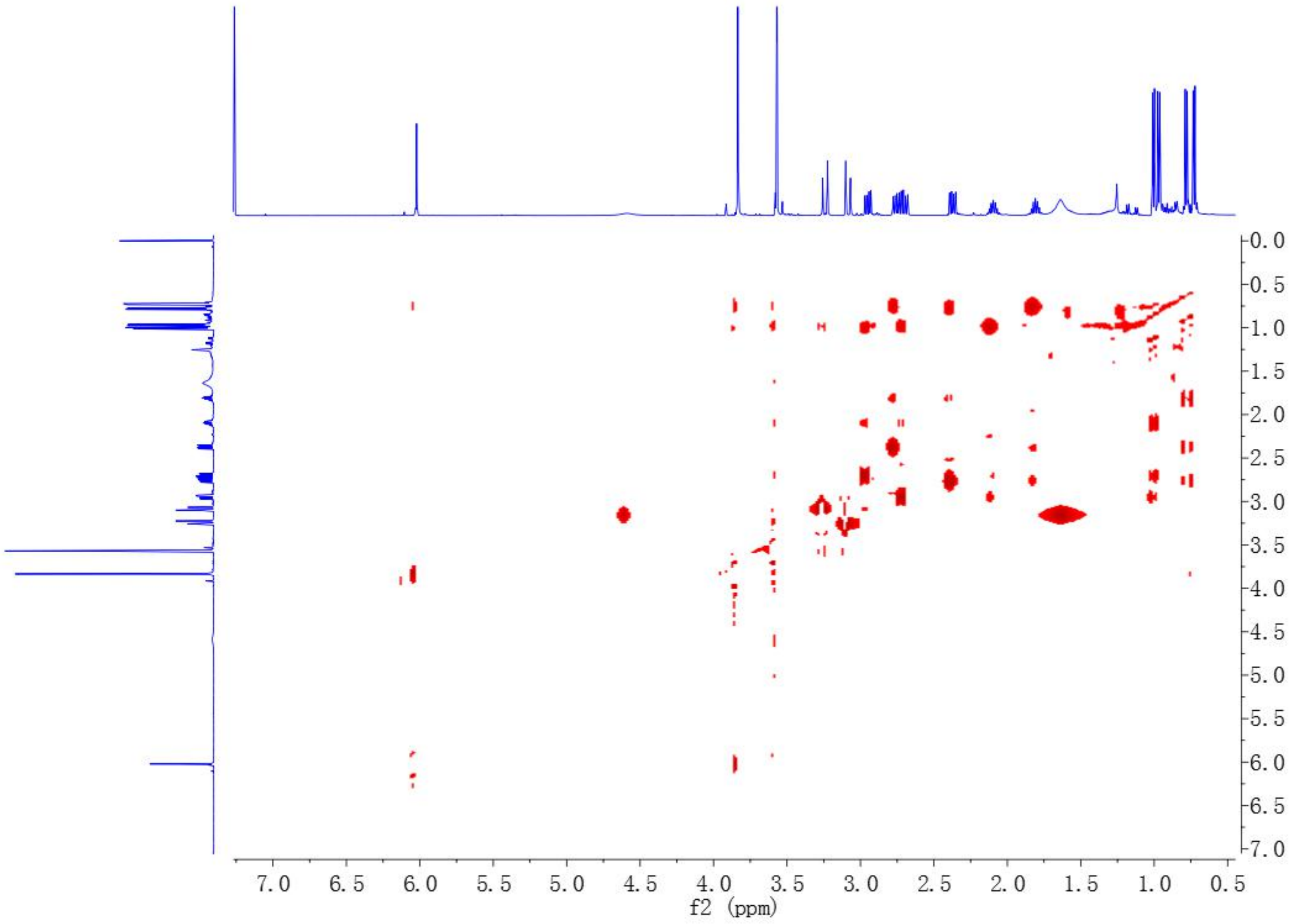

Parameter	Value (f2, f1)
1 Data File Name	C:/ HQC-1a/ 6/ pdata/ 1/ 2rr
2 Title	HQC-1a
3 Comment	
4 Origin	Bruker BioSpin GmbH
5 Owner	nmrsu
6 Site	
7 Spectrometer	spect
8 Author	
9 Solvent	CDCl3
10 Temperature	298.0
11 Pulse Sequence	noesygpphpp
12 Experiment	2D-NOESY
13 Number of Scans	8
14 Receiver Gain	147
15 Relaxation Delay	2.0000
16 Pulse Width	11.4200
17 Acquisition Time	0.1462
18 Acquisition Date	2015-06-17T21:50:08
19 Modification Date	2015-06-17T23:38:52
20 Spectrometer Frequency	(500.13, 500.13)
21 Spectral Width	(7002.8, 7002.8)
22 Lowest Frequency	(-100.5, -100.5)
23 Nucleus	(1H, 1H)
24 Acquired Size	(1024, 256)
25 Spectral Size	(1024, 1024)

Figure S7. NOESY spectrum of compound **1**.

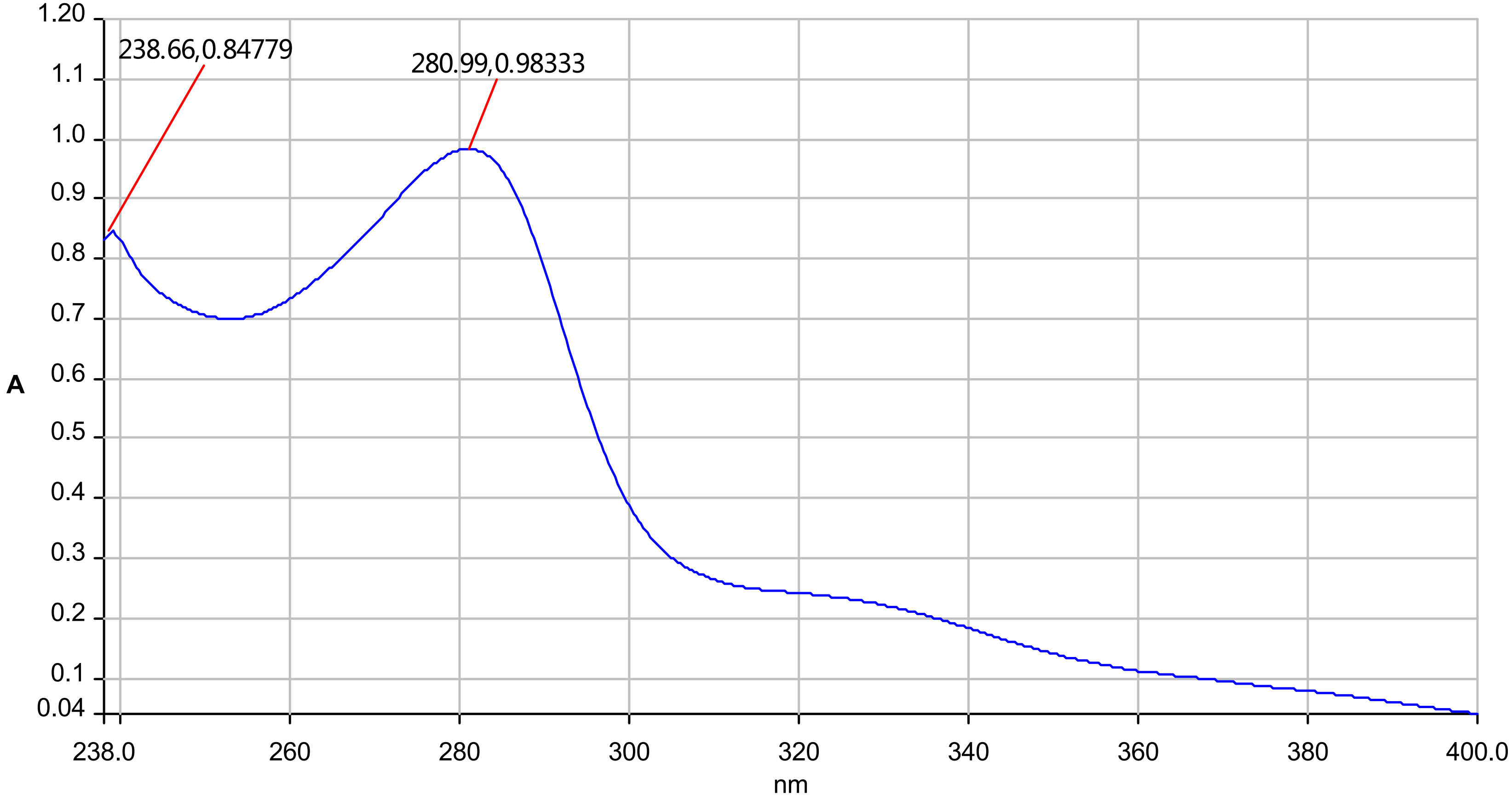

Figure S8. UV spectrum of compound **1**.
